# Can Task Specificity Impact tDCS-Linked to Dual Task Training Gains in Parkinson's Disease? A Protocol for a Randomized Controlled Trial

**DOI:** 10.3389/fnagi.2021.684689

**Published:** 2021-07-01

**Authors:** Adriana Costa-Ribeiro, Suellen Mary Marinho dos Santos Andrade, Mayane Laís Veloso Férrer, Ozair Argentille Pereira Da Silva, Maiara Llarena Silva Salvador, Suhaila Smaili, Ana Raquel Rodrigues Lindquist

**Affiliations:** ^1^NeuroMove Laboratory, Department of Physiotherapy, Federal University of Paraíba, Campus I Cidade Universitária, Joao Pessoa, Brazil; ^2^Neuroscience and Aging Laboratory, Federal University of Paraíba, Campus I Cidade Universitária, Joao Pessoa, Brazil; ^3^Laboratory of Intervention and Analysis of Movement, Department of Physiotherapy, Federal University of Rio Grande do Norte, Campus Universitário, Natal, Brazil; ^4^Department of Physiotherapy, State University of Londrina, Londrina, Brazil

**Keywords:** Parkinson's disease, transcranial direct current stimulation, physical therapy, dual-task, cognition, study protocol

## Abstract

Patients with Parkinson's disease (PD) have difficulties while performing dual-task activities, a condition present in everyday life. It is possible that strategies such as transcranial Direct Current Stimulation (tDCS) can be associated with motor training enriched with dual-task training to improve the performance of two concurrent tasks. Currently, it is unclear whether specific tasks and clinical conditions of PD patients have different results after the intervention. Therefore, the proposed randomized controlled trial will examine task-dependency in enhancing the effects of tDCS-linked rehabilitation training on PD and the relationships between baseline outcomes in responders and non-responders to therapy. Fifty-six patients with Parkinson's disease will be recruited to participate in this controlled, double-blind randomized multicentric clinical trial. Patients in modified Hoehn & Yahr stage 1.5–3, age between 40 and 70 years will be included. Subjects will be randomly assigned to an experimental group (EG) and a control group (CG). The EG will perform treadmill gait training associated with dual task exercises+tDCS, while the CG will only engage in treadmill gait training+tDCS. Blinded testers will assess patients before and after 12 intervention sessions and after a 4-week follow-up period. All patients will undergo a screening and an initial visit before being assessed for primary and secondary outcomes. The primary outcome measure is functional mobility measured by Timed Up and Go Test. Secondary outcomes include cognitive function, participation, motor function and body function and structure. This study will evaluate the effectiveness of an intervention protocol with tDCS, dual-task training and gait training in patients with PD. The study will also highlight the clinical factors and variability between individuals that could interfere in the training of a specific task and influence the therapeutic effect.

**Clinical Trial registration**: www.ClinicalTrials.gov, identifier NCT04581590.

## 1. Introduction

Parkinson's disease (PD) is a neurodegenerative disorder that affects motor and cognitive function, especially when individuals are submitted to dual tasks (Cameron et al., 2010; Kelly et al., 2012). During concomitant cognitive and motor tasks, the cerebral cortex prioritizes cognition, which may increase the risk of falls (Yarnall et al., 2011) and, consequently, of fractures and trauma (Duncan et al., 2011).

In PD, progressive cognitive impairment related to executive function, memory oscillation, language and visuospatial capacity may be present in the initial stages of the disease (Yarnall et al., 2014). Executive dysfunction occurs in 20% to 70% of cases (Elgh et al., 2009) and can help predict the occurrence of dementia in PD (Anang et al., 2014), with dysfunction in cognitive flexibility and operational memory (Domellöf et al., 2011) becoming more pronounced as the disease progresses (Ebersbach et al., 2013).

According to Vervoort et al. (2016), when compared to healthy individuals, those with PD exhibit changes in the brain connectivity of motor areas and the cerebellum when a dual task is required. Given the difficulty in executive function that these individuals face, dual-task training is recommended for this population (Brauer and Morris, 2010; Strouwen et al., 2014; Geroin et al., 2018). In addition, evidence shows the benefit of combined therapies to treat the disease (Zhou et al., 2014; Manenti et al., 2016; Vervoort et al., 2016), demonstrating improvement when patients with PD are submitted to cognitive training during gait while performing a dual task (Yogev-Seligmann et al., 2012). Among the non-pharmacological therapies is the association between transcranial direct current stimulation (tDCS) and rehabilitation protocols (Manenti et al., 2018; Beretta et al., 2020). It is suggested that synergic effects occur when both therapies are applied simultaneously, due to a likely modulation of the circuits that control planning and execute motor tasks (Fregni et al., 2006; Benninger et al., 2010; Kaski et al., 2014), with possible neuroplastic changes in the feedback loops that regulate the cognitive components also affected by PD (Manor et al., 2018).

tDCS is used to modulate cortical excitability, due to its action on neural membrane potential, leading to neuronal hyperpolarization via cathodic stimulation or depolarization provided by anodic stimulation (Nitsche and Paulus, 2000). Several studies show that in the rehabilitation of people with PD, tDCS has positive effects on motor function (Fregni et al., 2006), gait and bradykinesia (Benninger et al., 2010). When combined with physical training, tDCS can improve speed and balance during locomotion (Kaski et al., 2014) and seems to prolong the effects of motor intervention (Costa-Ribeiro et al., 2017).

Despite the promising results in reducing the symptoms of PD, the predictive factors that lead to a better response to intervention remain largely unknown. Disease severity, the medication dose in use, clinical type and freezing of gait seem to influence the effect and response to therapy. In relation to individual vulnerability, it is still unclear for whom these benefits apply, that is, whether age, disease severity or even emotional aspects linked to depression and anxiety before treatment determine treatment responsiveness (Carrarini et al., 2019). Moreover, the heterogeneity of the protocols used in the studies reflect the lack of understanding task-dependency associated with tDCS (Schoellmann et al., 2019). Which motor and cognitive components should be emphasized during tDCS-linked training has been little studied and whether multimodal protocols with mixed training should be prioritized over strictly motor or cognitive tasks.

In this clinical trial, we sought to investigate two aspects. First, to examine task-dependency in enhancing the effects of tDCS-linked rehabilitation training on PD. The relationships between baseline outcomes in responders and non-responders to therapy will also be examined, once, we also hypothesize that differences in clinical variables such as disease severity and depression symptoms may affect treatment effectiveness. In light of the evidence in previous studies (Swank et al., 2016; Manor et al., 2018), we hypothesize that a dual-task intervention program, including motor and cognitive paradigms, will be more robust in clinical measures than gait motor training alone.

## 2. Materials and Methods

### 2.1. Design

**Figure 1:**
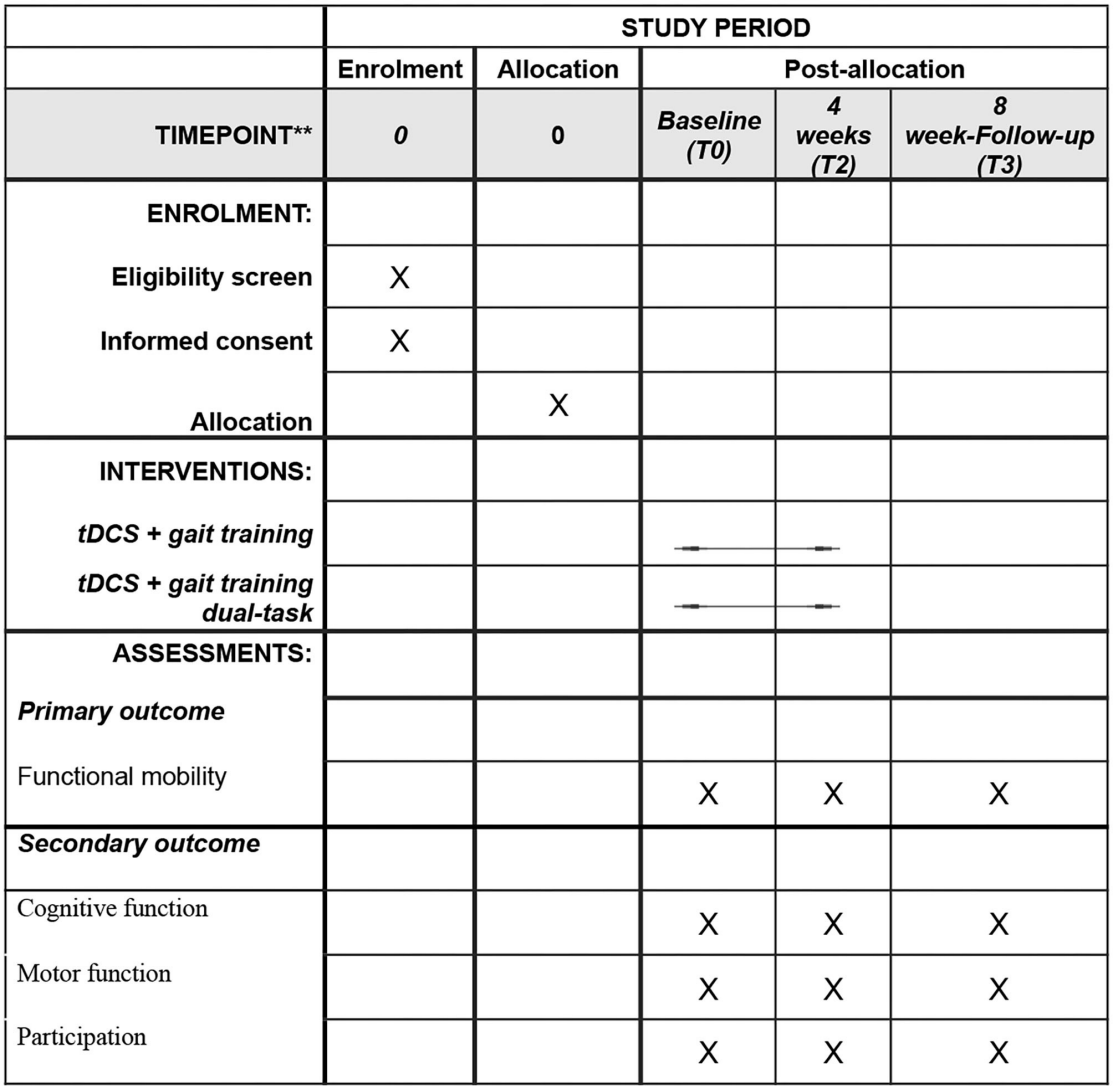
Schedule of enrollment, interventions and assessments demonstrated in the Standard Protocol Items: Recommendations for Interventional Trials (SPIRIT).

This is a controlled, double-blind randomized multicentric clinical trial, in line with the Standard Protocol Items Recommendations for Interventional Trials (SPIRIT) guideline (Chan, et al.), Figure 1. This project was approved by the Institutional Research Ethics Committee (30668420.7.0000.5188) and will be conducted according to the 1964 Declaration of Helsinki (Rickham, 1964). The trial was prospectively registered with the public platform clinical trials registry (www.clinicaltrials.org).

**Figure 2:**
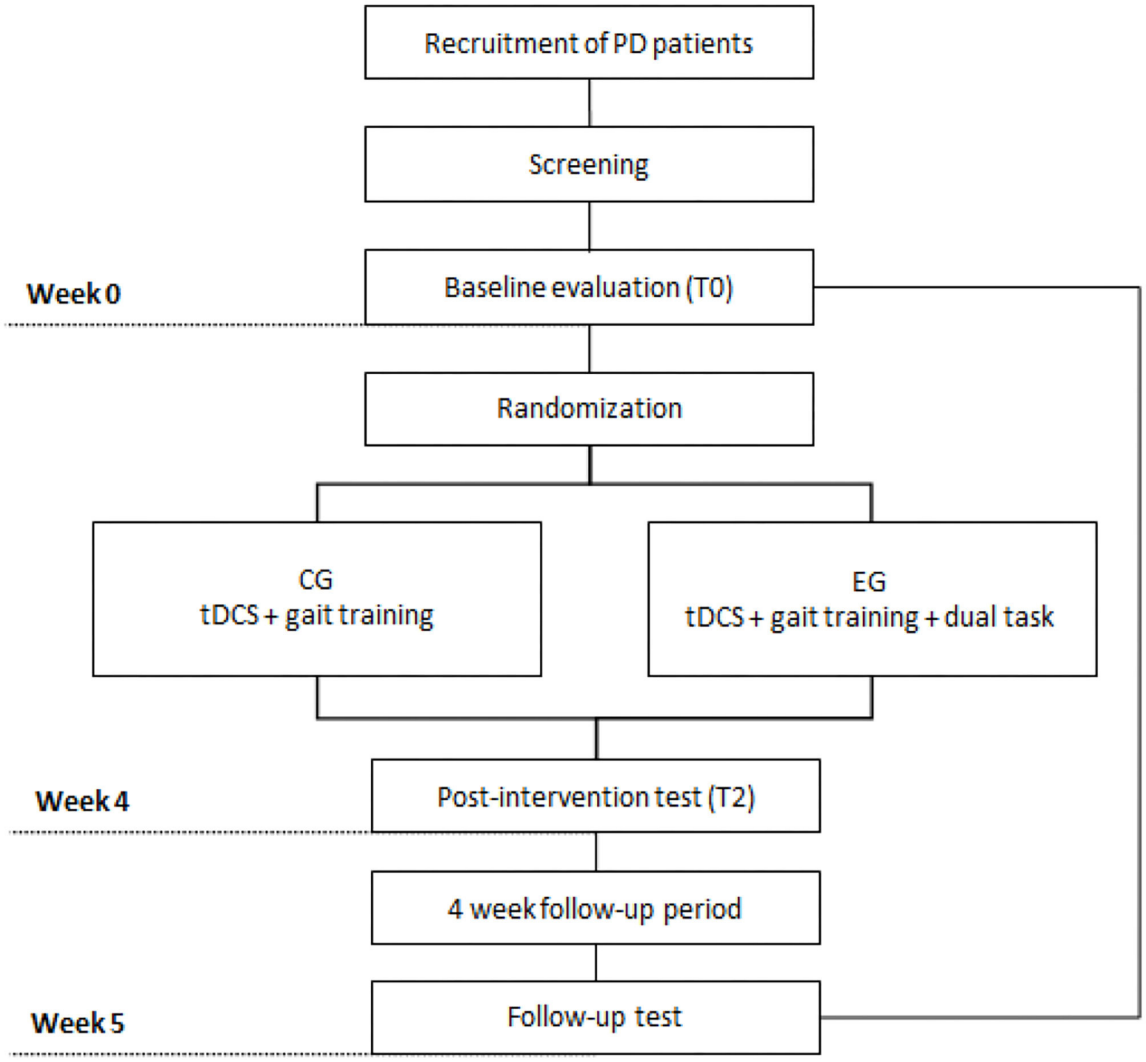
Design of the study. Legends: PD, Parkinson's disease, CG, Control Group, EG, Experimental Group, tDCS, transcranial direct current stimulation.

The treatment program for patients with PD will involve 12 sessions, three times a week. Study participants will be assessed at three different times: 4–7 days before the first intervention session, which will consider the baseline reference measure (T0); 4–7 days after the last session, considered a post-intervention measure (T1); and 30 days after the last intervention session, considered a post-intervention or follow-up measure (T2). Figure 2 illustrates the study design.

### 2.2. Participants

The following inclusion criteria will be applied: being diagnosed with idiopathic Parkinson's disease by a neurologist based on definitive evidence of responsiveness to levodopa at the start of the disease and the history of progressive hypokinesia with asymmetric onset. PD will be diagnosed based on Parkinson's Disease Society Brain Bank (PDSBB) criteria, as described in Hughes et al. (1992): age between 40 and 70 years, with no distinction for sex, schooling level or other sociodemographic characteristics; disease staging between 1.5 and 3, according to the modified Hoehn and Yahr scale (Hoehn and Yahr, 1998); undergoing regular pharmacological treatment with levodopa (equivalent dose > 300 mg) or taking antiparkinsonian medication, such as anticholinergics, selegiline, dopamine agonists, and COMT (catechol-O-methyl transferase) inhibitors for at least 4 weeks prior intervention; score of more than 24 points on the Mini-Mental State Examination (Folstein et al., 1975); not exhibiting other associated neurological diseases; and no musculoskeletal and/or cardiorespiratory changes that could compromise gait. The exclusion criteria will be diagnosis of atypical Parkinson's disease; neuropsychiatric comorbidities; convulsions, metal clips and/or pacemaker; deep brain stimulation implant; history of epilepsy; neurosurgery; traumatic brain injury; alcohol abuse or drug dependency; associated diseases of the peripheral or central nervous system; undergoing physical therapy at another location; inability to walk 10 meters; presence of important dyskinesia that prevents the participant from sitting in a chair; abnormal and persistent increase in systemic blood pressure before or during training, after three measurements taken 5 min apart-Cut-off: systolic blood pressure ≥ 140 mm Hg and/or diastolic ≥ 90 mm Hg (Malachias et al., 2016); not understanding any of the training protocol stages; chemical scalp treatment within the previous 30 days, and experiencing severe pain and/or discomfort that precludes performing the proposed activities.

History of falls in the last 12 months will be used to classify individuals into “non-fallers” (number of falls ≤ 1) or “recurrent fallers” (number of falls ≥ 2). Freezing of gait episodes will be screened and classified according to the dual-task screening questionnaire (Strouwen et al., 2014).

### 2.3. Recruitment

The multicentric study will be conducted at several centers that have specific units or not, which provide specialized assistance to patients with PD. Participants will be recruited from hospitals and clinics, as well as social media and support groups. The records of subjects interested in taking part will be analyzed and included in the study if they meet the eligibility criteria. All the individuals that agree to participate will provide written informed consent.

### 2.4. Randomization and Blinding

Participants will be randomly allocated, using an online generator (www.random.org), into two groups (1:1): CG) tDCS active + gait training; EG) tDCS active + gait training + dual-task. This sequence will be performed independently and remotely by a blinded investigator, who will have no knowledge of other study procedures. Randomization will be concealed until group allocation and stratified by subgroup with or without freezing of gait (FOG).

The concealed allocation process will be conducted using sequentially numbered sealed opaque envelopes. The outcome assessors, trialists and patients will be blinded to the procedures.

### 2.5. Attrition and Adherence

Attrition will be considered in case of: two consecutive absences or three alternate absences during the training sessions; changes in medication throughout the intervention; missing post-intervention or follow-up evaluations; illness that blocks continuity in the study. Adherence strategies will be used, such as telephone contact with participants, in order to remind them of the evaluation and intervention sessions. The hours offered will be flexible and possible problems that may interfere with the participation and continuity in the intervention will try to be prevented or resolved.

### 2.6. Screening

Study participants will be submitted to screening and an initial visit before being assessed for primary and secondary outcomes. A structured evaluation will be carried out, including sociodemographic data; time since diagnosis; disease severity according to the modified Hoehn and Yahr Clinical Staging Scale (Hoehn and Yahr, 1998; Goetz et al., 2004); medication doses used; levodopa equivalent daily dose (LEDD); symptoms of depression and anxiety, measured by the Hospital Anxiety Depression (HAD) scale; freezing of gait, assessed by the Freezing of Gait Questionnaire (FOG-Q) (Giladi et al., 2009); and type of disease, classified as akinetic-rigid, tremulant or mixed. Although of tremor-dominant and nontremor-dominant subtypes are the most comonly system used to defining Parkinson's disease subtypes, the participant's classification as postural instability gait disorder (PIGD) phenotype (or akinetic-rigid) has been used to specify a nontremor-dominant subtype (Marras, 2015). In this study, the participant's classification for PIGD score will be described based on “walking and balance” and “freezing” items of part II of Movement Disorder Society Unified Parkinson's Disease Rating Scale scores (MDS-UPDRS), and the “gait”, “freezing of gait” and “postural stability” items of part III of MDS-UPDRS scores (Jankovic et al., 1990). TD/PIGD scores will be used for all patients and calculate by dividing mean tremor subscores (2.10, 3.15a, 3.15b, 3.16a, 3.16b, 3.17a, 3.17b, 3.17c, 3.17d, 3.17 and 3.18) by mean PIGD subscores (2.12, 2.13, 3.10, 3.11 and 3.12) (Stebbins et al., 2013). Values equal to 1.15 classifiles the patient as TD subtype, whereas values equal to 0.90 represent PIGD. The patient will be classified as indeterminate subtype if the ratio between mean TD and mean PIGD valeus will be between 0.90 and 1.15 (Stebbins et al., 2013). The Mini-Mental State Examination (MEEM) will be applied in order to characterize the sample (Folstein et al., 1975). We will investigate lateral dominance aspects using the Edinburgh Handedness Inventory (Oldfield, 1971), and collect anthropometric data such as head circumference, inter-tragus and nasion-inion distance, height, weight and body mass index (BMI).

Screening will assess primary and secondary baseline outcomes. These assessments will be repeated at the endpoint (after the 12 sessions) and follow-up (after 1 month). The safety of tDCS application will be assessed at each session, by collecting information on perceived sensations, possible discomfort or adverse effects (Brunoni et al., 2011). All assessments will be conducted by physicians and physiotherapists with expertise in management of PD and application of specific scales for cognitive and motor assessment of people with PD.

### 2.7. Outcomes

The primary outcome will be functional mobility, measured using the Timed Up and Go test (Podsiadlo and Richardson, 1991), to stand up from a chair at the command: “Walk 3 meters, walk along a demarcated course, turn around and walk back to the chair, then sit down”. For secondary outcomes, the following instruments will be used to assess executive function: (1) Wisconsin Card Sorting Test (WCST) to assess planning, cognitive flexibility, working memory, monitorization, inhibition of perseverations, and aspects related to executive function (Heaton and Staff, 1993); (2) Stroop Test, to assess selective attention, inhibition, cognitive flexibility, processing speed, fluid intelligence and the semantic system (Lezak et al., 2004, Strauss et al., 2006); (3) Trail Making Test (TMT), to evaluate the ability to draw lines between consecutive numbers from 1 to 25 (Bowie and Harvey, 2006); (4) Verbal Fluency Test to assess semantic and phonemic fluency in 1 min (Brucki and Rocha, 2004); and (5) Montreal Cognitive Assessment(MoCA), to evaluate overall cognitive and executive function (Van Uem et al., 2016).

The following scales will be used to assess motor function: (1) Dynamic Gait Index (DGI), to assess eight aspects of gait as well as dynamic balance (Shumway-Cook et al., 2000); (2) 10-meter walk test, to estimate gait speed by recording the average time the patient takes to cover a distance of 10m in three attempts (Peters et al., 2013); (3) Borg Scale, to guide physical activity intensity level and measure perceived exertion during session training (Borg, 1982); (4) Sit-to-stand (STS) test; (5) Kinematics of Gait, to analyze movement using the Qualisys Motion Capture System (Qualisys Medical AB, 411 13, Gothenburg, Sweden). This system records the spatio-temporal variables of gait, as well as the angular variations of the hip, knee and ankle joints; (6) MiniBESTest, to measure aspects of static and dynamic balance (Franchignoni et al., 2010); (7) Revised Movement Disorder Society-Sponsored Revision of the Unified Parkinson's Disease Rating Scale (MDS-UPDRS), to assess motor function (Goetz et al., 2008); and (8) Short FES-I, to measure the level of confidence in performing daily activities, in addition to identifying fear of falling and possible social isolation (Kempen et al., 2008). All the instruments used to assess the primary and secondary outcomes are described in Table 1.

**Table 1:** Primary and secondary outcome measures.

	**Outcome measures**	**AV-1**	**AV-2**	**Follow-UP**
**Primary outcome measure**				
Fucntional mobility	Timed Up and Go Test (TUG)	X	X	X
**Secondary outcome measure**				
Cognitive function	Wisconsin Card Sorting Test (WCST)	X	X	X
	Stroop Test (ST)	X	X	X
	Trail Making Test (TMT)	X	X	X
	Verbal Fluency Test (VFT)	X	X	X
	Montreal Cognitive Assessment (MoCA)	X	X	X
Motor function	Dynamic Gait Index (DGI)	X	X	X
	Ten Meter Walk test (10MWT)	X	X	X
	Borg Scale	X	X	X
	Sit-to-stand Test (STS-5x)	X	X	X
	Kinematic gait variables	X	X	X
	MiniBESTest	X	X	X
	MDS-Unified Parkinson's Disease Rating Scale (MDS-UPDRS II and III)	X	X	X
Participation	ShortFES-I	X	X	X

### 2.8. Safety

In order to control adverse effects, patients will be asked about the sensations experienced during the session in terms of “tingling,” “burning,” “headache,” and “sleepiness” and other discomforts, which will be scored as intensity (1-none, 2-mild, 3-moderate, and 4-strong), and whether this effect is related to stimulation on a 5-point Likert scale (Brunoni et al., 2011); where, 1 represents no relation and 5 a strong relation. If any injury or strong discomfort is identified, therapy will be stopped and specialized medical assistance provided, at no cost to the participant. Any adverse effects will be documented along with symptom severity and duration, as well as the cause of the adverse effect.

### 2.9. Intervention

#### 2.9.1. tDCS

Patients will be submitted to 12 training sessions, 3 times a week, for 20 min (Bello and Fernandez-Del-Olmo, 2012), simultaneously to the rehabilitation program. Direct current (2 mA) will be transferred by a neurostimulator (Neuroelectrics Starstim eight system^®^), portable, battery operated, attached to the participant's body by means of a waist pack and positioned on the back to facilitate the monitoring and movement of the arms during treadmill training. Electrodes with dimensions of 5 × 7 cm (35 cm2) will be positioned on the scalp covered by sponges electrode soaked with 0.9% saline solution. The anodic electrode will be placed on F3 to stimulate the left dorsolateral prefrontal cortex, and the cathode electrode will be positioned over the right contralateral supraorbital frontal cortex (respectively F3 and Fp2 in the international 10–20 system Electroencephalography placement) (Homan et al., 1987; Antal et al., 2017; Lefaucheur et al., 2017). We chose this montage to match previous studies of tDCS in PD patients.

In order to verify the configuration of the electrode selected, the distribution and flow of the current for the tDCS configuration will be simulated using SimNIBS 2.1 (SimNIBS software, http://www.simnibs.org) and MNI (Montreal Neurological Institute) coordinates.

#### 2.9.2. Rehabilitation Program

The participants will perform 12–20 min treadmill gait training associated to dual task exercises+tDCS, 3 times a week (Experimental Group) or treadmill gait training+tDCS (Control Group). If patients exhibit altered vital signs, they will be asked to remain seated and try to relax. If the situation persists, the patient will be instructed to visit their physician.

#### 2.9.3. Dual-Task Training Protocol (DTTP)

The dual-task training protocol (DT) will consist in cognitive exercise categories: verbal fluency, mental screening tasks, discrimination, decision-making and reaction time tasks, which will be associated to treadmill gait training (Sousa et al., 2016). Verbal commands will focus on the following: (1) large strides; (2) heel strike; (3) raising the knees while walking (Kelly et al., 2012).

DT will be conducted using activities with three levels of difficulty (Tables 2–4), such as (i) mental screening tasks involving addition and subtraction, such as counting backwards from 100 and subtracting 3 or 7, while walking; (ii) verbal fluency tasks in which the participant is asked to name items that start with a particular letter or have a common characteristic (farm animals, words with the letter A, B, C, etc) while walking; (iii) discrimination and decision-making tasks, such as saying YES when the word “strawberry” is heard and NOTHING when no fruit is heard; (iv) fastening buttons and a zipper while walking; (v) Looking down and then up while walking; (vi) walking with head turns associated with motor or cognitive disorders; (vii) dual-task of carry a tray while walking (Mehrholz et al., 2015). Participants will only evolve from one level to the next when performance on the previous level is free of error. Thus, progression toward better performance of both the participant and the task will be based on the time that the participant will spend walking on the treadmill, i.e., increasing the walking speed or time on the treadmill in each block, performing the greatest number of words, presenting greater accuracy in decision-making, and reducing response time (Strouwen et al., 2014). At the end, participants will be asked to report the functional difficulties experienced during DT training.

**Table 2:** Dual-task training at difficulty level 1.

**Task**	**Task description**	**Outcome measurement**	**Task limitations**
Counting backwards from 100	Subject walks while counting backwards from 100	- Number of calculations concluded. - Number of incorrect calculations. - Gait speed.	Depending on ability, may be more difficult for some participants than others.
Walking while carrying a tray with only one empty glass	The subject walks while carry a tray with one empty glass	- Gait speed. - Number of stops.	Involving the upper limbs may affect gait pattern. Difficulty depends on the amount of water in the glass.
Naming items in general that start with a particular letter while walking (the participant chooses and indicates the category of the item to list)	The subject walks while naming items that start with a particular letter of any category chosen and indicated by the participant.	- Gait speed. - Number of words generated.	Depending on ability, may be more difficult for some participants than others.
Walking forward saying YES when they hear the word “strawberry”	Subjects walk while listening to a list of fruits and says “YES” when they hear the word “strawberry”.	- Gait speed. - Number of correct answers. - Number of errors.	Depending on ability, may be more difficult for some participants than others.
Getting keys and a wallet out of a pocket and change the pocket side.	Subject walks while moving objects from one pocket to another.	- Gait speed. - Number of stops.	Involving the upper limbs may affect gait pattern.
Fastening buttons and a zipper while walking	Subjects walks while Fastening a zipper.	- Number of calculations concluded - Number of incorrect calculations - Gait speed.	Depending on ability, may be more difficult for some participants than for others.
Looking from one side to another while walking	Subjects walk while turning their head from one side to another.	- Gait speed. - Range of head motion.	Head motion may change balance and gait pattern.

**Table 3:** Dual-task training at difficulty level 2.

**Task**	**Task description**	**Outcome measurement**	**Task limitations**
Counting backwards from 100 and subtracting 3 while walking.	Subjects walk while counting backwards from 100 and subtracting 3.	- Number of calculations concluded. - Number of incorrect calculations. - Gait speed.	Depending on ability, may be more difficult for some participants than others.
Walking while carrying a tray with at least one glass filled with water while say peron's names.	The subjects walks while carry a tray with at least one glass filled with water while say person's names.	- Gait speed. - Number of stops. - Amount of water spilled.	Involving the upper limbs may affect gait pattern. Difficulty depends on the amount of water in the glass.
Walking while naming items that have the same characteristics (marine animals, names of city, farm animals etc).	The subjects walks while naming items that have the same characteristics: farm animals.	- Gait speed. - Number of words generated.	Depending on ability, may be more difficult for some participants than others.
Walking forward saying “YES” when they hear the word “strawberry” and “No” when they hear the word “banana”.	Subjects walk while listening to a list of fruits and say “YES” when they hear the word “strawberry” and says “No” when hear the word “banana”.	- Gait speed. - Number of correct answers. - Number of mistakes.	Depending on ability, may be more difficult for some participants than others.
Getting a wallet out of pocket counting coins and moving them from one pocket to another.	Subjects walk while moving coins from one pocket to another	- Gait speed. - Number of correct answers. - Number of mistakes.	Depending on ability, may be more difficult for some participants than for others.
Fastening buttons and a count buttons while walking.	Subjects walk while fastening buttons and count buttons.	- Gait speed. - Number of buttons concluded.	Depending om ability, may be more difficult for some participants than for others.
Looking down and the up while walking and saying object names in general	Subjects walk while looking down and then up and sayings object names in general.	- Gait speed. - Number of correct objects.	Head movements may change balance and gait pattern mainly when damage the visio (lookink up).

**Table 4:** Dual-task training at difficulty level 3.

**Task**	**Task description**	**Outcome measurement**	**Task limitations**
Counting backwards from 100 and subtracting 7 while walking.	Subjects walk and move around obstacles while counting backwards from 100 and subtracting 7.	- Number of calculations concluded. - Number of incorrect calculations. - Gait speed.	Depending on ability, may be more difficult for some participants than others.
Walking while carrying a tray with at least two glasses filled with water while naming the cars models.	The subjects walk while carry a tray with at least two glasses filled with water while the naming cars models.	- Gait speed. - Number of stops. - Amount of water spilled. - Number of words named	Involving the upper limbs may affect gait pattern. Difficulty depends on the amount of water in the glass.
Naming items that have the same characteristics (farm animals with only two legs.	The subjects walks while naming items that have a common characteristics: farm animals with only two legs.	- Gait speed. - Number of words named. - Number of stops.	Depending on ability, may be more difficult for some participants than others.
Walking forward saying “YES” when they hear the word “watermelon” and say “No” when they hear all another fruit names pronounced by researcher	Subjects walks while listening to a list of fruits and say “YES” when they hear the word “watermelon” and “NO” when they hear all another fruit names pronounced by researcher.	- Gait speed. - Number of correct answers. - Number of stops. - Number mistakes	Depending on ability, may be more difficult for some participants than for others.
Getting a wallet out of a pocket, counting coins and moving them from one pocket to another.	Subjects walk while moving coins from one pocket.	- Gait speed. - Number of correct answers. - Number of mistakes.	Depending on ability, may be more difficult for some participants than for others.
Fastening buttons, count buttons and calculate the sum of the coins while walking.	Subjects walk while fastening buttons, count buttons and calculate the sum of the coins.	- Number of buttons concluded. - Gait speed.	Depending on ability, may be more difficult for some participants than for others.
Looking down and then up while walking and sayings words that begin with the letter “F”.	Subjects walk while and sayings words tha begin with the letter “F”.	- Gait speed. - Number of correct answers (words that begin the letter “F”).	- Head movements may change balance and gait pattern mainly when damage the vision (looking up).

### 2.10. Sample Calculation

The sample size power calculation was based on data from previous studies that used tDCS associated with motor training in people with PD (Manenti et al., 2016). The power calculations used to determine the number of participants in each group were made in relation to the expected change in functional mobility (primary outcome). Thus, a calculation considering *p* < 0.05 and 90% power as significant suggests that at least 46 patients would be necessary. Considering the possibility of sample losses throughout the study, we will aim to recruit 56 patients, totaling 28 participants per group.

### 2.11. Data Analyses

The Statistical Package for the Social Sciences (SPSS), version 27.0, will be used in data analysis and a 5% alpha (*P* < 0.05) will be established.

The groups will be compared using the Student's *t*-test or Mann-Whitney test, for continuous variables, or the chi-squared test for categorical variables, according to the normality distribution, analyzed using the Shapiro-Wilk test. The primary outcome will be examined with a repeated measures split-plot ANOVA, with one dependent and two independent variables: one intragroup (time, with 3 levels: T0, T1 and T2), and one intergroup (a group with two levels: CG and EG) using Sidak's *post hoc* test. Analyses of covariance (ANCOVA) will be used to identify significant intergroup differences applying T0 scores as covariables. Linear regression will be used to identify response predictors. The independent variables will be group and clinical response, that is, the minimal clinically important difference (MCID). An intention to treat analysis will be conducted.

## 3. Discussion

Progressive cognitive compromise in PD is considered a significant predictor of disability (Yogev-Seligmann et al., 2008) and an important cause for the decline in motor function (Lemes et al., 2016). This study aims at assessing the effectiveness of dual-task training and gait training conducted concomitantly with the neuromodulation protocol In addition, we intend to analyze the clinical factors and variability between individuals that could interfere in the training of a specific task and influence the therapeutic effect, acting as response predictors of the motor and cognitive function of people with PD.

### 3.1. Cortical Activity Shift in Parkinson's Disease

The preparation and selection of movement depends on the connective dynamics of the neural network between the prefrontal cortex and the lateral premotor cortex, compatible with the context-dependent role of the activity guided externally by the lateral premotor loop (Lemes et al., 2016). Greater activation of this loop in the executive function was demonstrated when the study of neuroimaging analyzed processing speed during the finger-tapping cognitive task in participants with Parkinson's disease in the off state (Palavra et al., 2013).

In individuals with PD, executive function (EF) deficiency, related to attention and a set-shifting change in focus is associated with gait dysfunctions when challenges involve a dual-task (DT). Given this condition (DT), neural network activation, consisting of the lateral premotor loop, becomes the compensatory attribution that externally guides the movement executed (Palavra et al., 2013).

### 3.2. Dual-Task and Its Relationshiap With Executive Function

The direct relationship between bradykinesia and mental flexibility and operational memory sustains the premise that EF in PD is correlated with motor function (Domellöf et al., 2011) and becomes a strong correlation as the disease progresses (Ebersbach et al., 2013).

Cognitive demand is considered a concurrent effort while people with PD and motor fluctuations walk (Hobson and Meara, 2004; Rochester et al., 2007; Plotnik et al., 2009; Michely et al., 2015). On the other hand, DT induces an increase in cholinergic activity in the dorsolateral prefrontal cortex, thalamus and basal ganglia (Yogev et al., 2005), which makes DT training important despite the greater risk of falling.

### 3.3. Relationship Between Executiva Function and Gait

Dysfunction in planning skills may result in poor choices and unnecessary effort for people with PD to reach a destination, once the functions linked to self-regulated processing, self-awareness and rational processes are mediated by the dorsolateral prefrontal cortex (Homan et al., 1987).

Complex cognitive tasks cause exponential harm to the gait parameters of this population (Plotnik et al., 2009), which makes these tasks relevant in changing the impact of cognitive demand on locomotion (Hackney and Earhart, 2010). Preparing meals and shopping in outdoor markets have an impact on self-awareness, and the ability to walk safely and efficiently, situations that characterize dysfunction in executive function (EF) (Yogev-Seligmann et al., 2008).

Cognitive training is an effective intervention strategy to improve the working memory, processing speed and EF of people with PD [64]. It is suggested that a training protocol be implemented using a clinical approach that increases EF in terms of memory and visuospatial function (Plotnik et al., 2011), as described in the dual task training proposed here.

The trail making test (TMT) keep a direct relationship between EF and gait speed/stride length changes (Hackney and Earhart, 2010). This test analyzes cognitive flexibility, demonstrating a direct association between complex gait situations and their scores in drawing lines that connect consecutive numbers and letters (Plotnik et al., 2011).

EF changes in PD can be identified using a series of tests, once no single test is able to predict subcortical dementia syndrome (Leung et al., 2015) as PD progresses. Abstract reasoning, measured using the Wisconsin card classification test (WCST) (Leung et al., 2015), requires an understanding of the logical principles of the problem (Nocera et al., 2010), and has shown to be a marker of executive dysfunction in PD (Beatty et al., 2003). Semantic memory, which is related to the ability of recalling memorized information and EF processing, with an emphasis on thought organization, is assessed using the verbal fluency test (Berg, 1948). Attention, screened by the speed in naming color words and the colors of incongruent words, is measured by the Stroop test (Lezak et al., 2004).

The difficulty in performing daily activities that require cognitive processing and simultaneous motor demand underscores the importance of DT training in improving the EF of people with PD (Brauer and Morris, 2010; Strouwen et al., 2014; Geroin et al., 2018).

In this respect, the dual-task locomotion test with the serial subtraction of 7 is useful (primary outcome) and will help define the executive function profile of the population under study. Other tests that analyze the effect of DT as a concurrent task on locomotion will contribute to the discussion of the data.

Combining tDCS as a rehabilitation intervention is a way to enhance motor training, given that modulation of the dorsolateral prefrontal cortex (DLPFC) may improve executive control (Lange et al., 2018). Applying anodic tDCS to F3 associated with cognitive training reduced the depressive symptoms reported by people with PD even after 3 months without intervention, demonstrating the effectiveness of cognitive training alone in increasing language, attention and executive function performance (Doruk et al., 2014; Manenti et al., 2018). However, it has yet to be established which patients respond better to noninvasive neuromodulation or how the association between different therapies, such as motor or cognitive training, responds when applied in conjunction with cortical modulation using tDCS (Rodrigues et al., 2008; Cools et al., 2010; Sale et al., 2015).

This is the first study that will compare the effect of two interventions associated with motor and cognitive tasks and noninvasive neuromodulation in a same treatment protocol. We hypothesize that after adding specific tasks that stimulate cognitive processing, the group that will undergo dual task training will exhibit improved cognition and motor function. Moreover, this study will contribute to better understanding neural substrates adjacent to cognitive training involved in the execution of the DT, and identify the response predictors of the proposed training, once it includes a detailed assessment of motor and cognitive aspects of patients with PD.

## Limitations and Advantages

Additional limitations should be noted. The estimated sample size may be limited. Nevertheless, our exploratory study is a step in the direction of large-scale studies. Furthermore, the study design enables secondary between-group analyses regarding baseline variables and predictors of treatment response. Finally, the study will provide robust results regarding the isolated and combined effects of tDCS and motor training.

## Ethics Statement

The studies involving human participants were reviewed and approved by Comitê de Ética em Pesquisa do Centro de Ciências da Saúde da Universidade Federal da Paraíba–CEP/CCS-(CAAE: 30668420.7.0000.5188; Parecer no:4.003.244). The patients/participants provided their written informed consent to participate in this study.

## Author Contributions

AC-R conceived the idea for the study, drafted the work, interpretation of sequence for the work, revising it critically for the text method and discussion. SA made substantial contributions for the text introduction and intellectual content of the present manuscript and contributed to the research design. AL made substantial contributions to the conception and design of the work and was critically important for the text discussion, interpretation of data for the work. MF showed substantial contribution in method topic elaboration, to the bibliographic research for definition of the protocol exercises, and data digitization in tables manuscript. OS performed the sample calculation, showed substantial contribution to the bibliographic research and was critically. All authors have made substantive intellectual contributions to the development of this protocol, and have read and approved the final protocol.

## Conflict of Interest

The authors declare that the research was conducted in the absence of any commercial or financial relationships that could be construed as a potential conflict of interest.
